# Impact of nucleos(t)ide analogues on the risk of hepatocellular carcinoma in chronic hepatitis B patients: a time-dependent Cox regression analysis

**DOI:** 10.3389/fgstr.2025.1585760

**Published:** 2025-06-03

**Authors:** Makoto Moriyama, Ryosuke Tateishi, Mizuki Nishibatake Kinoshita, Tsuyoshi Fukumoto, Tomoharu Yamada, Taijiro Wake, Ryo Nakagomi, Takuma Nakatsuka, Tatsuya Minami, Masaya Sato, Mitsuhiro Fujishiro, Kazuhiko Koike

**Affiliations:** ^1^ Department of Gastroenterology, Graduate School of Medicine, The University of Tokyo, Tokyo, Japan; ^2^ Kanto Central Hospital of the Mutual Aid Association of Public School Teachers, Tokyo, Japan

**Keywords:** CHB, chronic hepatitis B, hepatocellular carcinoma, nucleoside analogs, time-dependent covariate, cirrhosis

## Abstract

**Background and aims:**

The preventive effect of nucleos(t)ide analog (NA) use on HCC development in patients with chronic hepatitis B (CHB) is controversial due to the difficulty of conducting randomized controlled trials.

**Approach and results:**

In this single-center, retrospective study, NA-naïve CHB patients without a history of HCC were enrolled and followed-up from the first visit on or after January 2000 to December 2020. Patients were categorized into the NA group, including those who started NA after study enrollment, and the non-NA group, including patients who were never administered NA during the follow-up period. After propensity score matching (PSM) to balance the confounding factors, we applied a multivariable time-dependent Cox proportional regression analysis with the initiation of NA as a time-dependent covariate. We further performed a subgroup analysis according to the presence or absence of cirrhosis. The baseline characteristics of 212 pairs of patients retrieved by PSM were comparable. During the mean follow-up of 12.9 and 6.8 years in the NA and non-NA groups, respectively, 25 and 28 patients developed HCC, respectively. Multivariable analysis with time-dependent covariates showed that NA did not affect HCC risk (HR, 0.68; 95% CI, 0.36–1.31; *p* = 0.25) after adjusting for other risk factors, including age, sex, and HBV viral load. Subgroup analysis showed that NA use significantly reduced the risk of HCC in cirrhotic patients (HR, 0.26; 95% CI, 0.08–0.85; *p* = 0.03).

**Conclusions:**

The preventive effect of NA on hepatocarcinogenesis may be limited to cirrhotic patients.

## Introduction

Approximately 350 million people worldwide are chronically infected with HBV (1–3). Chronic hepatitis B (CHB) is a leading cause of liver-related adverse events, including liver cirrhosis and HCC. The annual incidence of HBV-related HCC varies according to risk factors, from <0.1% in health carriers to 2% to 5% in cirrhosis (4). Previous studies have reported that baseline HBV DNA load is a significant risk factor for HCC (5, 6).

Administration of nucleos(t)ide analogs (NAs) in CHB patients reduces viral load and suppresses liver fibrosis progression (7, 8). This suggests that the administration of NA in patients with CHB reduces HCC incidence. One randomized controlled trial supported this concept by comparing lamivudine administration to placebo in patients with cirrhosis and CHB, where HCC incidence assessed as a part of the composite outcome, was less frequent in the lamivudine group with a marginal significance (*p* = 0.047) (9). The unsustainable effect of lamivudine, due to the high rate of viral resistance development during long-term therapy, partially explains the reason for the marginal outcome (10, 11). Therefore, more potent NAs with lasting effects, including entecavir and tenofovir, may exert a more pronounced preventive effect on HCC development (12–14). As the beneficial effects of NA therapy on CHB have become increasingly evident, particularly in reducing the risk of hepatic decompensation, it is no longer ethically feasible to conduct randomized controlled trials specifically evaluating NA efficacy on hepatocarcinogenesis.

Several observational studies have shown that NA reduces the risk of NA on HCC (15–20). However, comparing treated and untreated groups reveals the following issues. First, if the observation period in the treated group started at the beginning of the treatment and at the patient’s first visit to the clinic in the untreated group, the latter has a substantially longer observation period. Second, if the observation began at the first visit to the clinic, and patients were divided into treated and untreated groups after enrollment, there is an immortal time bias: patients in the treated group will not experience the outcome during part of the follow-up period (**Supplementary Figure S1**) (21). To address these methodological challenges, statistical approaches such as Cox proportional hazard models with time-dependent covariates or landmark analysis are recommended (22, 23). This study aims to evaluate the effect of NA therapy on hepatocarcinogenesis in CHB patients using these robust statistical methods to overcome the limitations of previous observational studies.

## Patients and methods

### Study protocol

This retrospective study was conducted according to the ethical guidelines for epidemiological research established by the Japanese Ministry of Education, Culture, Sports, Science and Technology and Ministry of Health, Labour, and Welfare. The study design was included in a comprehensive protocol of retrospective studies at the Department of Gastroenterology, the University of Tokyo Hospital, and approved by the University of Tokyo Medical Research Center Ethics Committee (approval no. 2058). Informed consent was waived because of the retrospective design. The following statements were posted at a website (http://gastro.m.u-tokyo.ac.jp/patient/clinicalresearch.html) and participants who do not agree to the use of their clinical data can claim deletion of them.

### Patient and public involvement

Patients and/or the public were not involved in the design, or conduct, or reporting, or dissemination plans of this research.

### Data collection

We collected data from CHB patients diagnosed at our department since 1985, which was stored in a designated computerized database. We retrieved clinical data from the first visit after January 1, 2000 to December 31, 2020. These data included baseline characteristics: age, sex, presence of cirrhosis, and laboratory data, including total bilirubin, albumin, aspartate aminotransferase (AST), alanine aminotransferase (ALT), platelet count, HBeAg positivity, and HBV DNA load. The viral load was converted to IU/mL using the conversion formula described in **Supplementary Table S1**, as the measurement methods for HBV DNA changed during the study period. Cirrhosis was diagnosed based on clinical findings, laboratory data, imaging findings, and liver stiffness measured by transient elastography or liver biopsy. The database also stored the treatment regimen, the date of treatment initiation, and the response regarding NA use. NAs include lamivudine (LAM), adefovir (ADV), entecavir (ETV), tenofovir disoproxil fumarate (TDF), and tenofovir alafenamide (TAF).

### Patient enrollment

Inclusion criteria were as follows: (i) chronic infection with HBV, defined as being positive for hepatitis B surface antigen (HBsAg) for at least 6 months, and (ii) patients over 18 years of age. Exclusion criteria were as follows: (i) coinfection with chronic hepatitis C, (ii) history of HCC before enrollment, (iii) second opinion or referral cases without follow-up, (iv) those who received NA before study enrollment, and (v) those whose HBV DNA load was not measured at enrollment. The patients were divided into the NA group, which included patients who started NA after enrollment in the study, and the non-NA group, which included patients in whom NA was never administered during the follow-up period. According to the Japanese clinical practice guidelines for CHB (24), patients were recommended to receive NA with serum HBV DNA level >2–000 IU/mL and elevated ALT level (>31U/L), or with advanced fibrosis. However, 8 patients refused to start NA treatment despite meeting the criteria.

### Definition of viral response

Sustained virological response (SR) during NA treatment is defined as undetectable HBV DNA by a sensitive polymerase chain reaction (PCR) assay with a limit of detection according to the clinical practice guidelines for CHB (24, 25). The lower limit of detection sensitivity differs depending on the time of measurement and testing methods (**Supplementary Table S1**). Primary non-response was defined as less than one log_10_ IU/mL reduction of HBV DNA after 3 months of therapy. Virological breakthrough is defined as a confirmed increase in HBV DNA level of more than 1 log_10_ IU/mL compared to the lowest value HBV DNA level on-therapy.

### Follow-up and diagnosis of HCC

Patients were followed-up at the outpatient clinic with blood tests, including serum alpha-fetoprotein (AFP), *lens culinaris* agglutinin-reactive fraction of alpha-fetoprotein (AFP-L3), and des-gamma-carboxy-prothrombin (DCP) levels and ultrasonography at every 6 months according to Japanese clinical practice guidelines for HCC (26). Contrast-enhanced CT or MRI were performed when tumors were detected on ultrasonography or when tumor markers were elevated, suggestive of HCC. Patients were followed-up until any confirmed HCC diagnosis or the last visit before December 31, 2021. Data of patients who died from any cause without HCC diagnosis or who had a liver transplant were censored. Data fixation was performed on April 1, 2022.

The study endpoint was the development of HCC. Considering hyper-attenuation in the arterial phase and washout in the late phase as definite signs of HCC, a diagnosis of HCC was made by dynamic CT or MRI (27). When the imaging diagnosis was indeterminate, we confirmed HCC pathologically by ultrasound-guided tumor biopsy.

### Statistical analysis

Data are expressed as medians with 25^th^ to 75^th^ percentiles unless otherwise indicated. Numbers and percentages were used for qualitative variables. The categorical variables were compared with χ^2^ tests, ordinal variables were compared with the Cochran–Armitage test, and continuous variables were compared with unpaired Student’s *t*-tests. To normalize the two groups (NA and non-NA groups), propensity score matching (PSM), including age, sex, presence of cirrhosis, total bilirubin, albumin, AST, ALT, platelet count, HBeAg positivity, and HBV DNA >2–000 IU/mL at baseline, was performed. In PSM analysis, we used logistic regression to estimate the probability of a patient to start NA treatment and generated a propensity score for each patient. Caliper matching on the propensity score was performed, and pairs were matched within a range of 0.2 of the standard deviation of the logit of the propensity score. The cumulative incidence of HCC was assessed with the Kaplan–Meier method. For the estimation of cumulative incidence of HCC with time-dependent grouping, we used the Simon and Makuch method, which was a computational method to graphically represent survival curves for time-dependent covariates (28). We also estimated the prognosis after HCC development with the Kaplan–Meier method.

We performed univariable and multivariable Cox proportional regression analyses using time-fixed and time-dependent covariates in the PSM cohort. Exposure to NA in addition to albumin, ALT, platelet count, and HBV DNA load were treated as time-dependent covariates. We further performed a subgroup analysis according to the presence or absence of cirrhosis and HBeAg status. Additionally, we conducted a comparative analysis of HCC risk between patients receiving ETV, TDF, or TAF versus those receiving LAM to evaluate the impact of NA potency on hepatocarcinogenesis.

We also performed 1-year and 2-year landmark analyses in the PSM cohort to mitigate the immortal time bias, where patients were stratified according to NA use prior to the corresponding time points.

Statistical analyses were performed using the R software (Ver. 4.1.3; R Development Core Team, Vienna, Austria). All tests were two-tailed. *p*-values < 0.05 were considered statistically significant.

## Results

### Patient profiles

**Figure 1:**
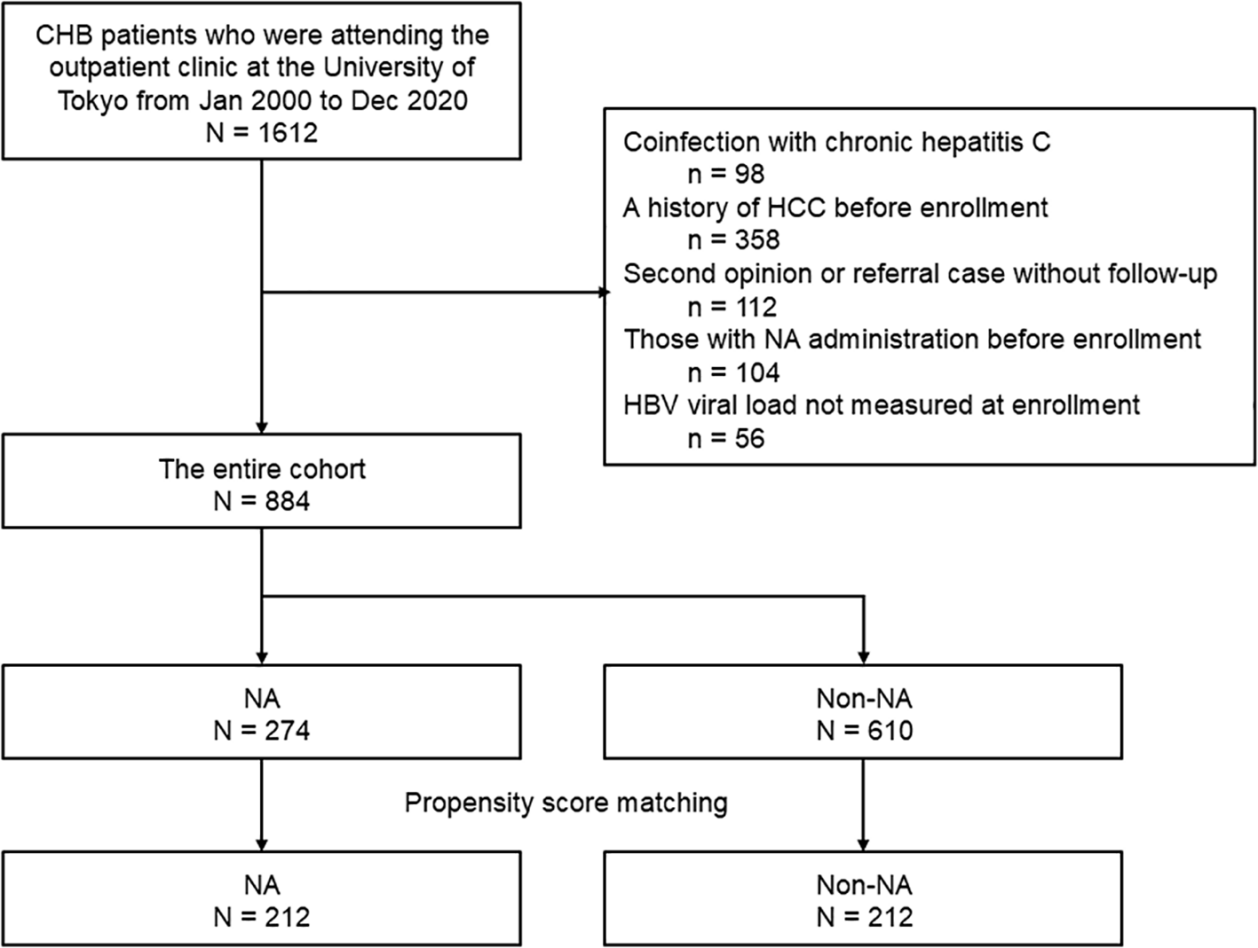
Patient enrollment flow.

Among the 1612 patients with CHB identified using a database search, 884 fulfilled the enrollment criteria (**Figure 1**). A total of 274 patients started receiving NA after enrollment (NA group) and 610 patients did not receive NA (non-NA group). The median interval from the study enrollment to the initiation of NA therapy was 2.19 (0.35-5.66) years, and the median duration of NA administration was 7.83 (3.80–12.0) years. The baseline characteristics of the entire cohort and the matched cohort are shown in **Table 1**. Significant differences were found in terms of sex, presence of cirrhosis, total bilirubin levels, albumin levels, AST, ALT, platelet count, the proportion of those with HBeAg positivity, and HBV DNA>2–000 IU/mL. The NA group included a higher proportion of patients with HBeAg, presence of cirrhosis, and high viral HBV load. The baseline characteristics of the 212 pairs of matched patients from the two study groups were comparable.

**Table 1:** Baseline characteristics of the entire cohort (N = 884) and Propensity score-matched cohort (N = 424)*.

	Entire cohort			Propensity score-matched cohort		
Variable	NA (n = 274)	Non-NA (n = 610)	*p*	NA (n = 212)	Non-NA (n = 212)	*p*
Age, years	49.0 (36.0–59.0)	48.0 (36.0–58.0)	0.92	48.0 (36–57)	47.0 (33–60)	0.98
Male sex	195 (71.2)	355 (58.2)	<0.01	143 (67.5)	153 (72.2)	0.34
Cirrhosis	58 (21.2)	47 (7.7)	<0.01	38 (17.9)	34 (16.0)	0.70
Total bilirubin, mg/dL	0.9 (0.7–1.0)	0.7 (0.6–1.0)	<0.01	0.8 (0.7–1.0)	0.8 (0.6–1.0)	0.97
Albumin, g/dL	4.1 (3.8–4.3)	4.3 (4.1–4.5)	<0.01	4.15 (3.9–4.4)	4.2 (4.0–4.3)	0.57
AST, IU/L	47 (31–84)	24 (19–39)	<0.01	42 (28–72)	36.0 (24–60)	0.70
ALT, IU/L	57.0 (32–129)	26 (17–47)	<0.01	53.5 (30–117)	44.5 (24–92)	0.95
Platelet count, × 10^4^/µL	17.4 (12.7–22)	20.6 (17.2–24)	<0.01	18.2 (13.4–23)	18.8 (15.1–22)	0.92
HBeAg positive	152 (55.5)	140 (23.0)	<0.01	101 (49.3)	111 (54.1)	0.85
HBV-DNA > 2–000 IU/mL	249 (90.9)	275 (45.1)	<0.01	188 (88.7)	185 (87.3)	0.77

*Values are presented as medians (interquartile ranges) or numbers (%).

The t-test test for continuous variables and the chi-squared test for categorical variables were used to compare them between the groups.

AST, aspartate aminotransferase, ALT, alanine aminotransferase.

### Nucleic acid analogs and viral response

A total of 274 patients received NAs during the study period. The treatment pathways are illustrated in **Supplementary Figure S2** as a Sankey diagram, demonstrating the flow of patients between initial and subsequent therapies. Initial treatment consisted of lamivudine (LAM) in 74 patients (27.0%), entecavir (ETV) in 182 patients (66.4%), tenofovir disoproxil fumarate (TDF) in 5 patients (1.8%), and tenofovir alafenamide (TAF) in 13 patients (4.7%). Among LAM-initiated patients, 25 patients (33.8%) transitioned to LAM+ Adefovir (ADV) combination therapy, 25 (33.8%) switched to ETV monotherapy, 5 (6.8%) changed to TAF, and 19 (25.7%) had unassessable subsequent treatment. Viral breakthrough was observed in 31 LAM patients, and primary non-response in 1 patient. For ETV-initiated patients, 179 patients (98.4%) continued ETV therapy. Two patients (1.1%) switched to TAF, and 1 patient (0.5%) had an unassessable treatment course. Viral breakthrough was observed in 3 ETV patients. All TDF-initiated patients (n=5) transitioned to TAF therapy during the follow-up period. All TAF-initiated patients (n=13) continued TAF therapy without requiring treatment modification. No viral breakthrough was observed in either TDF or TAF groups. In total, 271 of 274 NA-treated patients (98.9%) achieved sustained virological response by the end of the observation period.

### HCC development

**Figure 2:**
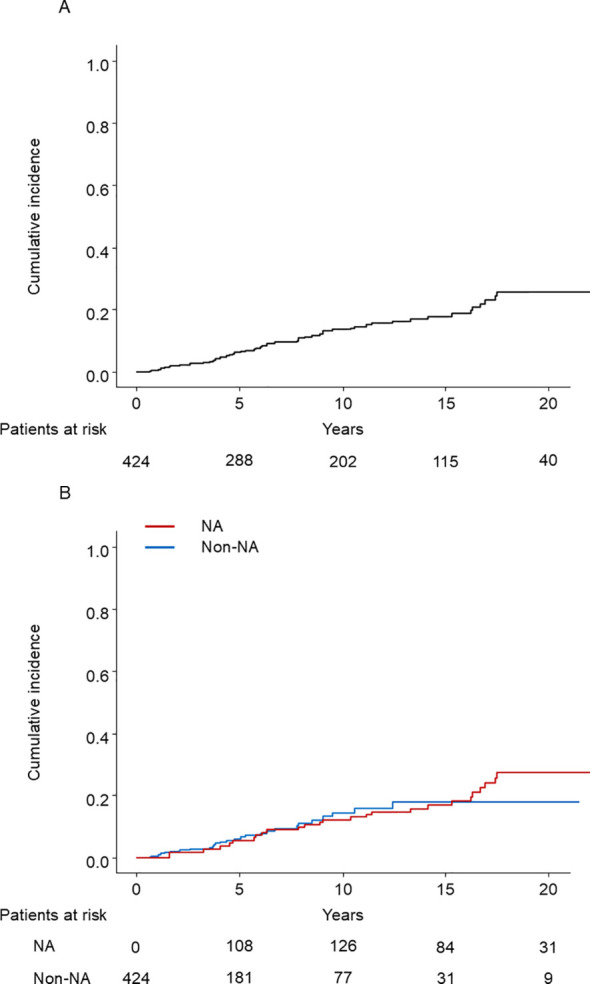
**(A)** Kaplan–Meier estimate of cumulative incidence of HCC in the PSM cohort (N = 424). HCC, hepatocellular carcinoma; PSM, propensity score matching. **(B)** The cumulative incidence of HCC with time-dependent grouping plotted by the Simon and Makuch method. HCC, hepatocellular carcinoma; NA, nucleos(t)ide analog.

In the entire cohort, 78 patients developed HCC: 38 in the NA group and 40 in the non-NA group. In the PSM cohort, during the mean follow-up of 12.9 and 6.8 years in the NA and non-NA groups, respectively, 25 patients of the NA group and 28 of the non-NA group developed HCC. The cumulative incidence rates of HCC development by Kaplan–Meier analysis at 5 and 10 years were 6.0%, and 12.7%, respectively (**Figure 2A**). The survival curves determined for NA use and non-use (time-dependent covariate) using the Simon and Makuch method are shown in **Figure 2B** (28).

HCC incidence rates stratified by HBeAg status revealed significant differences, with 5-year and 10-year cumulative incidence rates of 6.9% and 13.0% in HBeAg-positive patients versus 4.4% and 7.4% in HBeAg-negative patients, respectively (p = 0.007) (**Supplementary Figure S3**).

The characteristics of HCC are shown in **Table 2**. HCC was diagnosed at an earlier stage, i.e., with smaller size and fewer nodules, in the NA group than in the non-NA group. AST and ALT at the diagnosis of HCC were significantly lower in the NA group than in the non-NA group.

**Table 2:** Characteristics of HCC patients at diagnosis in the propensity score-matched cohort (N = 53)*.

Variable	NA (n = 25)	Non-NA (n = 28)	*p*
Age, years	69 (54-74)	60 (54-67)	0.08
Tumor size, mm	20 (16–25)	22 (20.5–30.0)	0.03
Number of nodules			0.09
Solitary	23 (92.0%)	19 (67.9%)	
2–3	1 (4%)	8 (28.6%)	
>3	1 (4%)	1 (3.6%)	
AFP >15 ng/mL	3 (12%)	12 (42.9%)	0.02
AFP-L3>10%	1 (4%)	7 (25%)	0.07
DCP>100 mAU/mL	6 (24%)	5 (17.9%)	0.89
Total bilirubin (mg/dL)	0.8 (0.6–1)	0.8 (0.6–1.0)	0.57
Albumin (g/dL)	4.0 (3.9–4.3)	4.0 (3.6–4.2)	0.21
AST (IU/L)	28 (19–31)	41 (28.5–47)	0.01
ALT (IU/L)	19 (14–27)	38 (22–51)	0.01
Platelet count (× 10^4^/µL)	17 (13–20)	12.8 (10.5–18)	0.19

*Values are presented as medians (interquartile ranges) or numbers (%).

The t-test for continuous variables, the chi-squared test for categorical variables, and the Cochran–Armitage test for ordinal variables were used to compare the groups.

AFP, alpha-fetoprotein, AFP-L3, lens culinaris agglutinin-reactive fraction of AFP, DCP, des-gamma-carboxy prothrombin, AST, aspartate aminotransferase, ALT, alanine aminotransferase.

### Prognosis after HCC development

Among 53 patients who developed HCC, 21 (5 in the NA group and 16 in the non-NA group) died before the end of the observation period. The median survival time after HCC development was 16.5 years. The survival curves after HCC development according to the NA use at the diagnosis were shown in **Supplementary Figure S4**. The log-rank test showed no significant difference between the two groups (*p* = 0.3).

### Univariable and multivariable time-dependent Cox regression analysis after PSM

The univariable analysis showed that the following factors were significantly associated with HCC development: age, presence of cirrhosis, albumin level, AST>40 IU/mL, and platelet count. In the univariable analysis, the HR of NA use was 1.10 (95% CI, 0.61–1.98; *p* = 0.75). In the multivariable analysis, NA use did not significantly decrease the risk of HCC (HR, 0.68; 95% CI, 0.36–1.31; *p* = 0.25) (**Table 3**).

**Table 3:** Univariable and multivariable analyses of hepatocarcinogenesis in CHB patients (Propensity Score-Matched cohort).

Variable	Univariable		Multivariable	
	HR (95% CI)	*p*	HR (95% CI)	*p*
Age per 1 year*	1.05 (1.03–1.07)	<0.01	1.05 (1.02–1.08)	<0.01
Female sex*	0.87 (0.47–1.61)	0.67	0.68 (0.37–1.27)	0.36
Cirrhosis*	5.27 (3.06–9.08)	<0.01	4.10 (2.25–7.49)	<0.01
Total bilirubin (mg/dL)^†^	1.04 (0.90–1.20)	0.64		
ALB per 1 g/dL^†^	0.42 (0.29–0.61)	<0.01	0.77 (0.48–1.24)	0.28
AST>40 IU/mL^†^	2.94 (1.54–5.61)	<0.01		
ALT>40 IU/mL^†^	1.81 (0.96–3.38)	0.07	1.74 (0.89–3.40)	0.11
Platelet count (× 10^4^/µL)^†^	0.91 (0.88–0.95)	<0.01		
HBeAg positive*	0.88 (0.51–1.52)	0.65	1.20 (0.67–2.14)	0.54
HBV-DNA >2–000 IU/mL^†^	1.47 (0.58–3.68)	0.42	1.49 (0.58–3.84)	0.41
NA use^†^	1.10 (0.61–1.98)	0.75	0.68 (0.36–1.31)	0.25

* These variables were analyzed as time-fixed covariates.

^†^ These variables were analyzed as time-dependent covariates.

AST, aspartate aminotransferase; ALT, alanine aminotransferase; NA, nucleos(t)ide analog.

### Subgroup analysis by cirrhosis status

**Figure 3:**
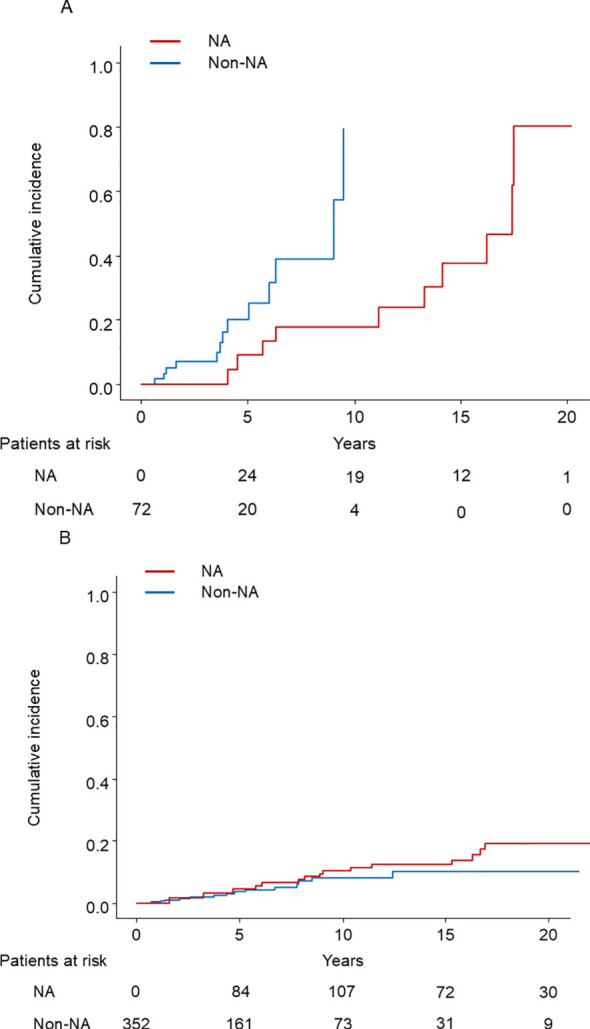
**(A)** The cumulative incidence of HCC in cirrhotic patients with time-dependent grouping plotted by the Simon and Makuch method. HCC, hepatocellular carcinoma; NA: nucleos(t)ide analog. **(B)** The cumulative incidence of HCC in noncirrhotic patients with time-dependent grouping plotted by the Simon and Makuch method. HCC, hepatocellular carcinoma; NA, nucleos(t)ide analog.

The PSM cohort was divided into 72 cirrhotic and 352 noncirrhotic patients, each analyzed according to the presence or absence of cirrhosis. HCC developed in 23 (10 in the NA group and 13 in the non-NA group) and 32 (17 in the NA group and 15 in the non-NA group) of cirrhotic and noncirrhotic patients, respectively. The survival curves determined for NA use and non-use using the Simon and Makuch method are shown in **Figures 3A, B**. There was a significant difference between the use and non-use of NA in cirrhotic patients (*p =* 0.04), but not in non-cirrhotic patients (*p* = 0.32). The results of univariable and multivariable Cox proportional regression analyses using time-fixed and time-dependent covariates are shown in **Tables 4**, **5**. In multivariable Cox proportional hazards analysis adjusted for time-fixed and time-dependent covariates, NA use significantly reduced the risk of HCC (HR, 0.26; 95% CI, 0.08–0.85; p=0.03) only in cirrhotic patients.

**Table 4:** Univariable and multivariable analyses of hepatocarcinogenesis: A subgroup analysis in cirrhotic patients.

Variable	Univariable		Multivariable	
	HR (95% CI)	*p*	HR (95% CI)	*p*
Age per 1 year*	1.04 (1.01-1.08)	0.02	1.05 (1.01-1.10)	0.03
Female sex*	0.70 (0.30-1.64)	0.41		
Total bilirubin (mg/dL)^†^	0.85 (0.61-1.19)	0.36		
ALB per 1 g/dL^†^	0.75 (0.41-1.36)	0.34		
AST>40 IU/mL^†^	1.20 (0.41-3.56)	0.74		
ALT>40 IU/mL^†^	1.02 (0.42-2.46)	0.97		
Platelet count (× 10^4^/µL)^†^	0.99 (0.92-1.07)	0.87		
HBeAg positive*	1.86 (0.82-4.20)	0.14	2.21 (0.90-5.43)	0.08
HBV-DNA>2–000 IU/mL^†^	3.46 (0.47-25.6)	0.23	3.06 (0.38-24.6)	0.29
NA use^†^	0.30 (0.10-0.93)	0.04	0.26 (0.08-0.85)	0.03

* These variables were analyzed as time-fixed covariates.

^†^ These variables were analyzed as time-dependent covariates.

AST, aspartate aminotransferase, ALT, alanine aminotransferase.

**Table 5:** Univariable and multivariable analyses of hepatocarcinogenesis: A subgroup analysis in noncirrhotic patients.

Variable	Univariable		Multivariable	
	HR (95% CI)	*p*	HR (95% CI)	*p*
Age per 1 year*	1.05 (1.02-1.08)	< 0.01	1.05 (1.02-1.08)	< 0.01
Female sex*	0.70 (0.28-1.71)	0.43		
Total bilirubin (mg/dL)^†^	0.92 (0.59-1.43)	0.73		
ALB per 1 g/dL^†^	0.44 (0.26-0.75)	< 0.01	0.58 (0.30-1.12)	0.10
AST>40 IU/mL^†^	2.67 (1.18-6.05)	0.02		
ALT>40 IU/mL^†^	2.37 (0.96-5.89)	0.06	2.95 (1.10-7.92)	0.03
Platelet count (× 10^4^/µL)^†^	0.96 (0.90-1.02)	0.17		
HBeAg positive*	0.55 (0.25-1.18)	0.12		
HBV-DNA>2–000 IU/mL^†^	0.94 (0.33-2.70)	0.91	0.90 (0.30-1.12)	0.85
NA use^†^	1.47 (0.67-3.23)	0.34	0.75 (0.31-1.79)	0.52

* These variables were analyzed as time-fixed covariates.

^†^ These variables were analyzed as time-dependent covariates.

AST, aspartate aminotransferase, ALT, alanine aminotransferase.

### Subgroup analysis by NA type

**Figure 4:**
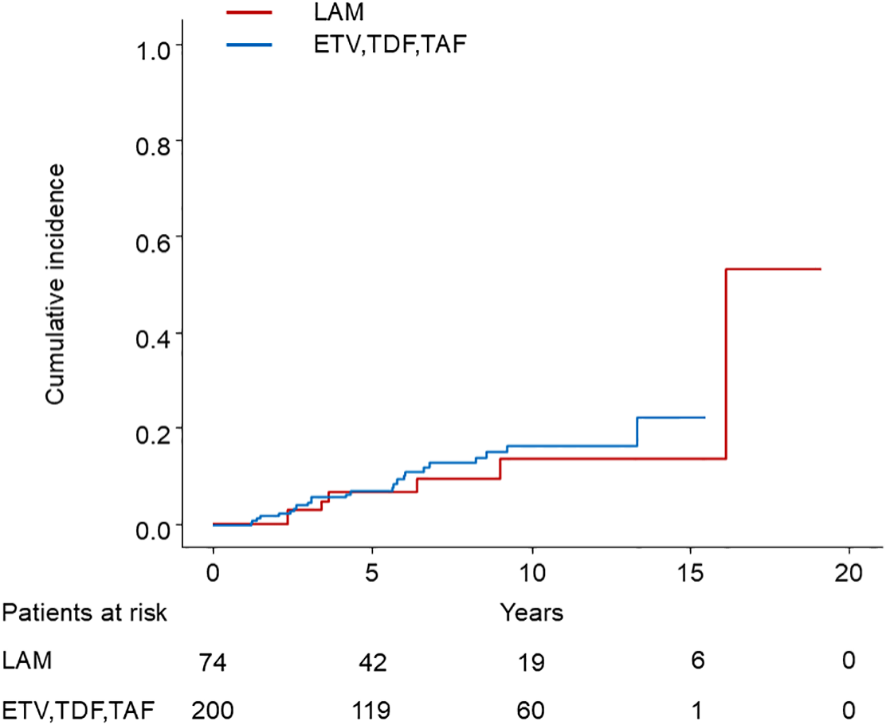
Comparison of HCC incidence between LAM and ETV, TDF, or TAF. HCC, hepatocellular carcinoma; LAM, Lamivudine; ETV, Entecavir; TDF, Tenofovir disoproxil fumarate; TAF, Tenofovir alafenamide.

Among 75 patients who started treatment with LAM, 7 developed HCC, while among 199 patients who started treatment with ETV, TDF, or TAF, 24 developed HCC. Patients receiving ETV, TDF, or TAF showed no statistically significant difference in HCC incidence compared to those receiving LAM (p = 0.4) (**Figure 4**).

### Landmark analysis

**Figure 5:**
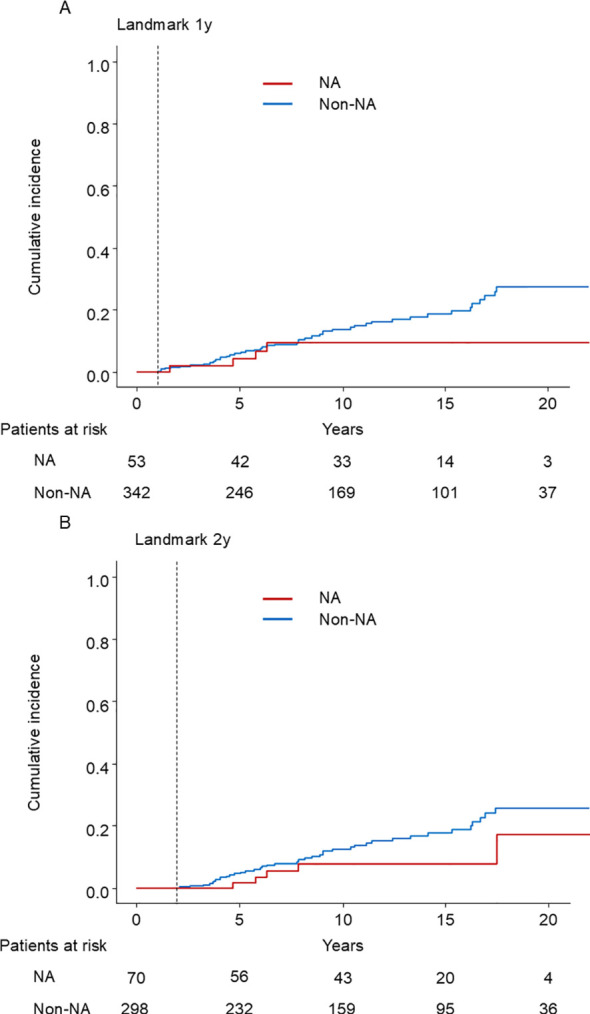
Kaplan–Meier estimate of cumulative incidence of HCC. **(A)** 1-year landmark analysis **(B)** 2-year landmark analysis NA: nucleos(t)ide analog.

We also performed 1-year and 2-year landmark analyses adjusting for age, sex, presence of cirrhosis, albumin, ALT, and HBV DNA load in the multivariable Cox proportional hazard model. Among the 395 patients who were under observation beyond 1 year after enrollment, 53 patients started NA treatment within 1 year. In the cohort, 51 patients developed HCC (4 patients with NAs and 47 without NAs) (**Figure 5A**). The HR of the NA was 0.47 (95% CI, 0.17–1.34; *p* = 0.16). Among the 368 patients who were under observation beyond 2 years after enrollment, 70 patients started NA treatment. 45 patients developed HCC (5 patients with NAs and 40 without NAs) (**Figure 5B**). The HR of the NA was 0.43 (95% CI, 0.16–1.11; *p* = 0.08). To conclude, NA treatment did not significantly reduce the risk of HCC.

## Discussion

To the best of our knowledge, this is the first study to evaluate the effect of NA on hepatocarcinogenesis in CHB patients using the time-dependent Cox regression analysis. Our study demonstrated that NA use did not significantly affect overall HCC risk in the total cohort of chronic hepatitis B patients, while subgroup analysis revealed a significant preventive effect specifically in patients with cirrhosis.

Previous observational studies have reported more favorable carcinogenic inhibitory effects of NA, which differs from our findings in the total cohort (15–20). A cohort study with PSM by clinical background showed that ETV use reduced the risk ratio of carcinogenesis to 0.37 (15). A retrospective cohort study of 1870 patients reported a lower 5-year carcinogenesis in patients with cirrhosis, with a 0.55 risk ratio in the ETV-treated group compared to the historical control group (19). One potential explanation for the discrepancy is treatment initiation criteria for NA, which could act as a confounding factor. Patients expected to have a worse prognosis tend to be initiated on NA therapy, which could explain why NA use was paradoxically correlated with increased risk of HCC in our univariable analysis, which became insignificant in the multivariable analysis after adjusting for other significant risk factors. This suggests that previous studies may have overestimated the tumor preventive effect of NA due to insufficient adjustment for confounding factors. Indeed, the incidence of hepatitis B-associated HCC has not changed over the past two decades, despite NA being widely available in Japan (29).

Various factors are reported to contribute to HBV-induced carcinogenesis, such as accumulation of genetic abnormalities in the process of repeated hepatocyte necrosis and regeneration due to chronic inflammation, the induction of mutations and increased genomic instability due to incorporation of HBV DNA into the host genome, and direct action of HBV X protein, which is expressed by HBV (32–35). NA can suppress chronic inflammation by regulating HBV replication; however, it may not suppress the integration of HBV DNA into the host genome or the direct action of HBV X protein. Therefore, it may be difficult to completely inhibit carcinogenesis with NA.

In papers with subgroup analyses by presence or absence of cirrhosis, NA therapy reduced the incidence of hepatocellular carcinoma only in patients with cirrhosis, except for one paper (36). Hosaka et al. reported that HCC incidence was decreased in ETV-treated patients compared to untreated patients in cirrhosis (*p* < 0.01), whereas no significant difference was observed in noncirrhosis (*p*= 0.44) (15). In a nationwide cohort study conducted in Taiwan, Wu et al. reported NA therapy was associated with decreased HCC incidence (20). However, the impact was smaller in noncirrhosis than in cirrhosis (HR, 0.72 vs. 0.27). Our subgroup analysis also showed that NA therapy reduced the HCC incidence only in cirrhotic patients. It may take a longer duration to observe decreased HCC incidence in noncirrhotic patients.

Although the incidence of HCC did not differ between the two groups, HCC was diagnosed at an earlier stage in the NA group, which might suggest the presence of lead-time bias. However, the lead time was not long, considering the difference in mean tumor size was only 2 mm. Another explanation is that the tumor doubling time was longer in the NA group with reduced signals of tumor progression propagated by necroinflammation, supported by the lower AST and ALT (37).

Some reports indicated that entecavir, which yields higher potency for viral suppression than lamivudine, could suppress the HCC incidence more effectively (15, 30, 31). However, in our study, patients who started treatment with ETV, TDF, or TAF showed no statistically significant difference in HCC incidence compared to those who started treatment with LAM. This is because even though 42% of patients who initiated LAM therapy experienced viral breakthrough, all but those who were lost to follow-up were subsequently switched to LAM+ADV, ETV, TDF, or TAF which ultimately provided effective viral suppression.

There were some limitations to this study. First, this is a retrospective study. Second, although we performed PSM, we could not completely exclude selection bias, as the use of NA is recommended for those at higher risk of HCC according to clinical practice guidelines. Third, because the unit of DNA differs depending on the time of measurement, we had to set a single cut-off point for HBV DNA by converting different units according to the conversion formula. More precise analysis using continuous variables may yield a different result.

In conclusion, the preventive effect of NA against liver carcinogenesis may be limited to patients with cirrhosis.

## Data Availability

The raw data supporting the conclusions of this article will be made available by the authors, without undue reservation.

## Supplementary material

Supplementary Figure 1 Schematic presentation of the immortal time bias. **Scenario 1**: Patients are divided according to the use of nucleos(t)ide analogs (NAs) at baseline. Patients 3 and 4 may have benefited from NAs even though they are categorized in the non-NA group. **Scenario 2**: Patients are divided according to the use of NAs during the observation period. There is the immortal bias: Patients 3 and 4 are guaranteed to be HCC-free up to the point of NA initiation.

Supplementary Figure 2 Treatment transitions among nucleos(t)ide analogs (NAs) in chronic hepatitis B patients. This Sankey diagram visualizes how patients transitioned between different NAs during the study period. The left side shows initial treatments (LAM, ETV, TDF, and TAF), while the right side displays subsequent therapies. The width of each flow represents the proportion of patients following each treatment pathway. LAM, Lamivudine; ADV, Adefovir; ETV, Entecavir; TDF, Tenofovir disoproxil fumarate; TAF, Tenofovir alafenamide.

Supplementary Figure 3 Cumulative incidence of HCC according to HBeAg status. HCC, hepatocellular carcinoma.

Supplementary Figure 4 Kaplan-Meier curve for overall survival after HCC development. HCC, hepatocellular carcinoma; NA: nucleos(t)ide analog.
