# Supplementary figures and images for: Impact of nucleos(t)ide analogues on the risk of hepatocellular carcinoma in chronic hepatitis B patients: a time-dependent Cox regression analysis
Source: Front Gastroenterol (Lausanne). 2025 Jun 3;4:1585760. doi: 10.3389/fgstr.2025.1585760 (PMC12952401; doi:10.3389/fgstr.2025.1585760)

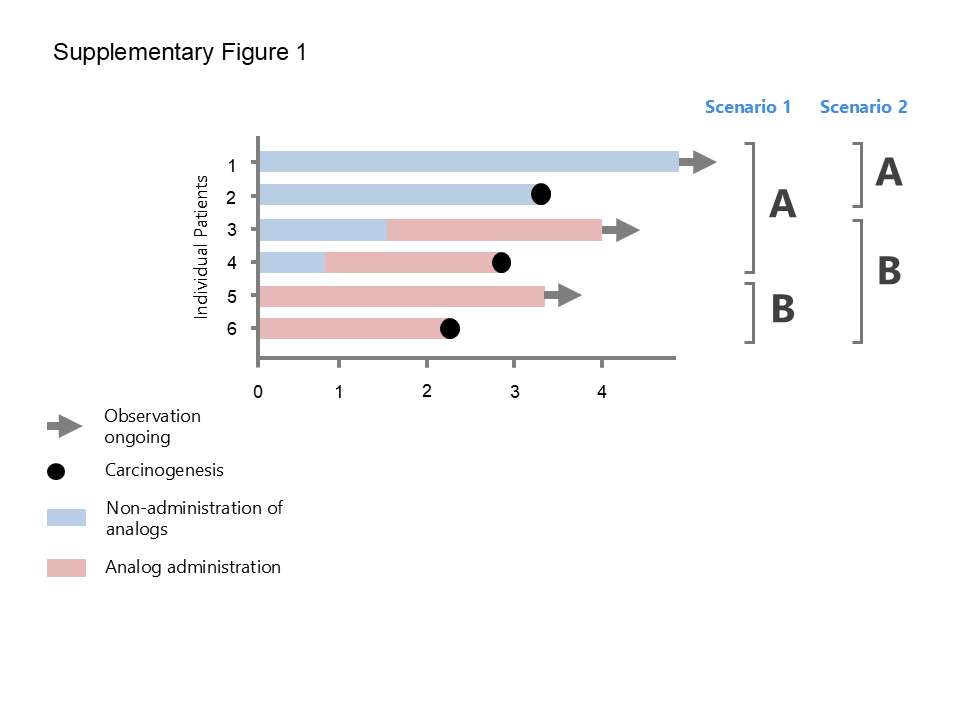

Supplement: Supplementary Figure 1 — Schematic presentation of the immortal time bias. Scenario 1: Patients are divided according to the use of nucleos(t)ide analogs (NAs) at baseline. Patients 3 and 4 may have benefited from NAs even though they are categorized in the non-NA group. Scenario 2: Patients are divided according to the use of NAs during the observation period. There is the immortal bias: Patients 3 and 4 are guaranteed to be HCC-free up to the point of NA initiation. [file Image1.tif]

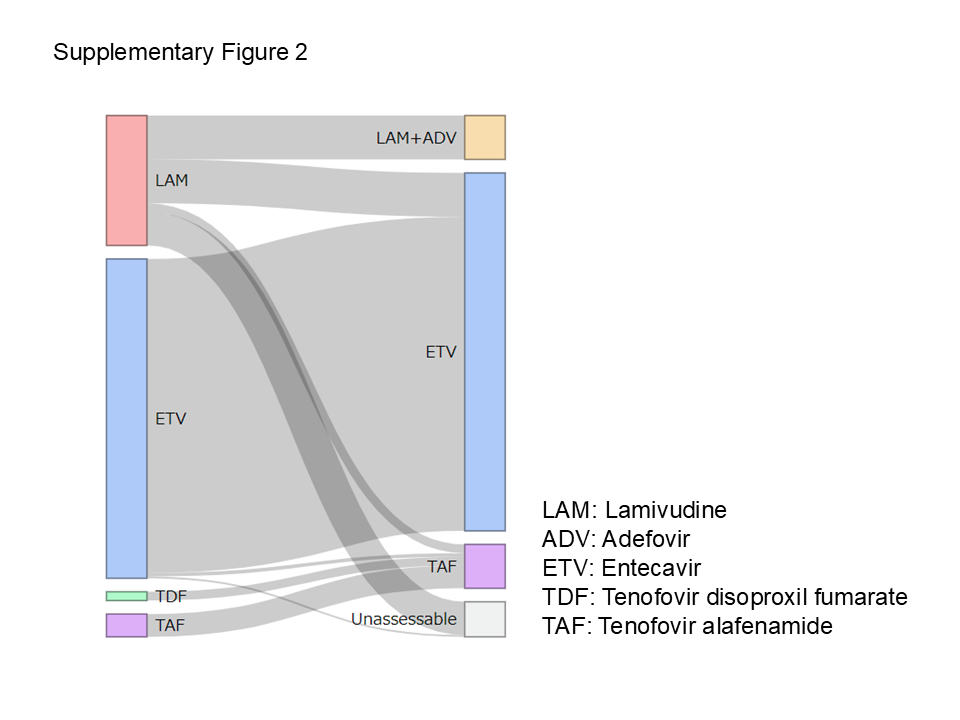

Supplement: Supplementary Figure 2 — Treatment transitions among nucleos(t)ide analogs (NAs) in chronic hepatitis B patients. This Sankey diagram visualizes how patients transitioned between different NAs during the study period. The left side shows initial treatments (LAM, ETV, TDF, and TAF), while the right side displays subsequent therapies. The width of each flow represents the proportion of patients following each treatment pathway. LAM, Lamivudine; ADV, Adefovir; ETV, Entecavir; TDF, Tenofovir disoproxil fumarate; TAF, Tenofovir alafenamide. [file Image2.tif]

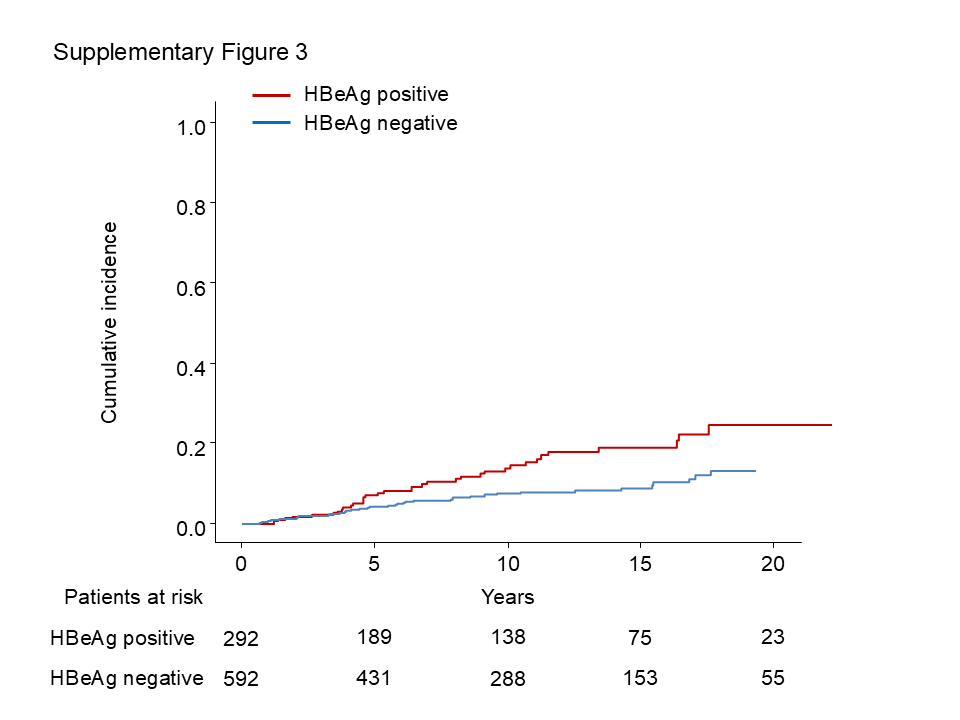

Supplement: Supplementary Figure 3 — Cumulative incidence of HCC according to HBeAg status. HCC, hepatocellular carcinoma. [file Image3.tif]

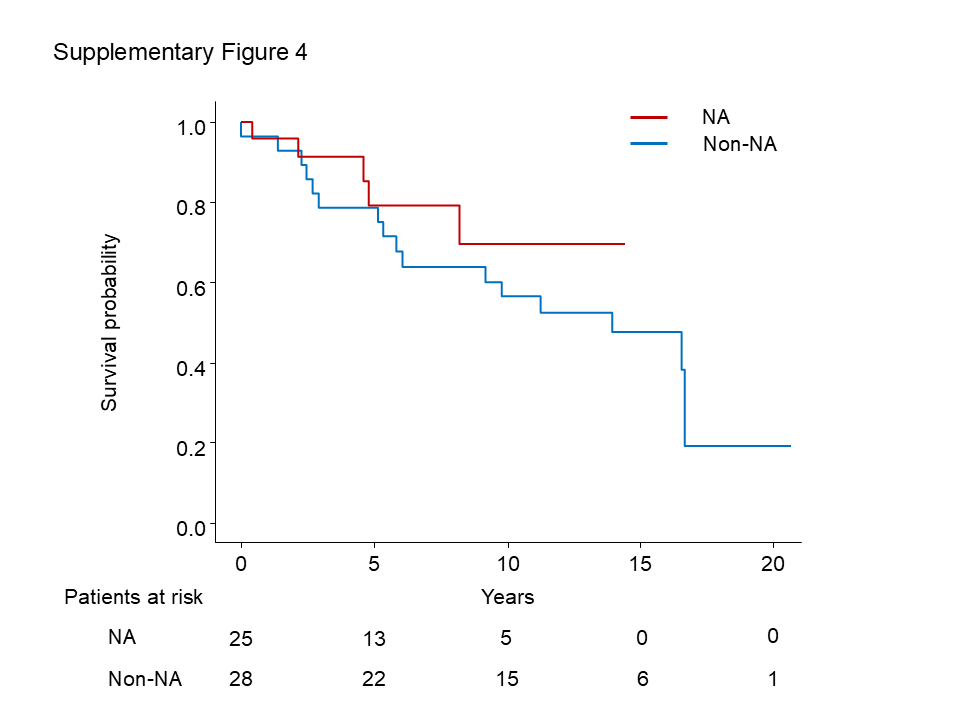

Supplement: Supplementary Figure 4 — Kaplan-Meier curve for overall survival after HCC development. HCC, hepatocellular carcinoma; NA: nucleos(t)ide analog. [file Image4.tif]
