# Supplementary material for: Impact of nucleos(t)ide analogues on the risk of hepatocellular carcinoma in chronic hepatitis B patients: a time-dependent Cox regression analysis
Source: Front Gastroenterol (Lausanne). 2025 Jun 3;4:1585760. doi: 10.3389/fgstr.2025.1585760 (PMC12952401; doi:10.3389/fgstr.2025.1585760)
Supplement: Supplementary file 5 [file DataSheet1.pdf]

Supplementary Table 1. Hepatitis B Virus Assay Method

Period	Measurement method	Unit	Lower limit	Upper limit
From January 1, 2000 to March 1, 2007	TMA-HPA	LGE/mL	3.7	8.7
From March 2, 2007 to December 31, 2016	Amplicor PCR	log copy/mL	2.6	7.4
From January 1, 2016	Real-time PCR	log IU/mL	1.53	9.5
